# The Effect of Body Temperature Changes on the Course of Treatment in Patients With Pneumonia and Sepsis: Results of an Observational Study

**DOI:** 10.2196/52590

**Published:** 2024-03-01

**Authors:** Domen Guzelj, Anže Grubelnik, Nina Greif, Petra Povalej Bržan, Jure Fluher, Žiga Kalamar, Andrej Markota

**Affiliations:** 1 Faculty of Medicine University of Maribor Maribor Slovenia; 2 Faculty of Electrical Engineering and Computer Science University of Maribor Maribor Slovenia; 3 Medical Intensive Care Unit University Medical Centre Maribor Maribor Slovenia

**Keywords:** fever, targeted temperature management, pneumonia, sepsis, intensive care unit

## Abstract

**Background:**

Traditionally, patients who are critically ill with infection and fever have been treated with antipyretics or even physically cooled. Presumed benefits of the reduction of body temperature are mostly based on decreased metabolic demands. However, it has been shown that decreasing body temperature in patients who are critically ill is not associated with improvement in treatment outcomes. Additionally, there is some data to support the use of temperature modulation (therapeutic hyperthermia) as an adjuvant treatment strategy in patients with infection.

**Objective:**

This study aims to determine the effect of body temperature on the course of intensive care unit (ICU) treatment of patients who are mechanically ventilated with pneumonia, sepsis, and positive tracheal aspirates on admission.

**Methods:**

We performed a single-center retrospective study. Core body temperature was measured in all patients. We analyzed associations between average temperatures in the first 48 hours after admission to ICU and ICU treatment parameters. Additionally, patients were divided into three groups: patients with negative tracheal aspirates 1 week after ICU admission (P-N group), patients with a different pathogen in tracheal aspirates 1 week after ICU admission (P-HAP group), and patients with a persisting pathogen in tracheal aspirates 1 week after ICU admission (P-P group). Differences in body temperature and interventions aimed at temperature modulation were determined.

**Results:**

We observed a significantly higher average temperature in the first 48 hours after admission to ICU in patients who survived to hospital discharge compared to nonsurvivors (mean 37.2 °C, SD 1 °C vs mean 36.9 °C, SD 1.6 °C; *P*=.04). We observed no associations between average temperatures in the first 48 hours after ICU admission and days of mechanical ventilation in the first 7 days of treatment (ρ=–0.090; *P*=.30), the average maximum daily requirement for noradrenaline in the first 7 days of treatment (ρ=–0.029; *P*=.80), average maximum FiO_2_ in the first 7 days of ICU treatment (ρ=0.040; *P*=.70), and requirement for renal replacement therapy in the first 7 days of ICU treatment (mean 37.3 °C, SD 1.4 °C vs mean 37.0 °C, SD 1.3 °C; *P*=.23). In an additional analysis, we observed a significantly greater use of paracetamol in the P-N group (mean 1.0, SD 1.1 g vs mean 0.4, SD 0.7 g vs mean 0.4, SD 0.8 g; *P*=.009), a trend toward greater use of active cooling in the first 24 hours after ICU admission in the P-N group (n=11, 44% vs n=14, 33.3% vs n=16, 32%; *P*=.57), and no other significant differences in parameters of ICU treatment between patient groups.

**Conclusions:**

We observed better survival in patients who developed higher body temperatures in the first 48 hours after admission to the ICU; however, we observed no changes in other treatment parameters. Similarly, we observed greater use of paracetamol in patients with negative tracheal aspirates 1 week after ICU admission. Our results support the strategy of temperature tolerance in patients who are intubated with pneumonia and sepsis.

## Introduction

Increased body temperature has been recognized as a sign of illness for more than 2000 years and antipyretics have been used for at least 100 years with the aim of lowering body temperature in patients who are febrile [1]. However, a body of evidence exists ranging from studies in the preantibiotic era to a recently published pilot trial, where increased body temperature is investigated as a treatment option in patients with infection [2-4]. A fever-range increase of body temperature is a highly preserved response that is probably beneficial in patients who develop fever as a part of a normal immune response to infection [5]. Recently, we have observed that patients with lower body temperatures are at a higher risk of acquiring the presence of multidrug-resistant pathogens [6]. The aim of this study was to investigate the association between body temperature and the course of intensive care unit (ICU) treatment in patients with pneumonia and sepsis and to evaluate the effect of temperature on the persistence of positive control tracheal aspirates.

## Methods

### Study Design and Settings

We performed retrospective observational data collection from January 1, 2018, to December 31, 2021. The study was performed in a medical ICU in a tertiary center and supported by an institutional research grant (grant IRP-2022/01-01).

### Ethical Considerations

The study was approved by University Medical Centre Maribor Ethical Committee, and informed consent was waived because of the observational and retrospective nature of the study (No. UKC-MB-KME-35/22). Patients’ data have been anonymized and deidentified. No compensation was provided to the participants.

### Study Population

We included adult (aged >18 years) patients with pneumonia, septic shock, positive tracheal aspirates within 24 hours after admission (“admission” tracheal aspirates), and tracheal aspirates withdrawn 8-14 days after admission to ICU (“week 2” tracheal aspirates). We excluded patients who were treated with targeted temperature management for accidental hypo- or hyperthermia, patients after cardiac arrest, and patients who are neurocritical (eg, patients with meningitis, encephalitis, ischemic or hemorrhagic stroke, or subarachnoid hemorrhage). Temperature management in our patient cohort was as per the treating physician.

### Measurements

We collected basic demographic data and data related to ICU treatment, namely outcome of ICU treatment, ICU length of stay, core body temperature during ICU stay, use of renal replacement therapy, use of acetaminophen, maximum concentration of noradrenaline, maximum fraction of inspired oxygen, maximum level of positive end-expiratory pressure and maximum minute ventilation, and microbiological results of tracheal aspirates. Source data was paper based for temperature and therapeutic charts and electronic for other data. Core body temperature was used for study data. As per department policy, temperature was measured via thermal probe urinary catheters (Rüsch Sensor Urinary Catheters, Teleflex Medical, Athlone, Ireland), and temperature measurements were continuously displayed on ICU monitors (Philips IntelliVue MX800 Patient Monitoring System, Koninklijke Philips N.V., Amsterdam, Netherlands). If insertion of a urinary catheter is not possible, then an esophageal temperature probe is inserted, but temperature measurement in all of the included patients was performed via a urinary catheter. Temperature results from 2-hourly notations were used for statistical analysis. We compared the association between body temperature and course of treatment parameters between three groups of patients: patients with sterile week 2 tracheal aspirates (P-N group), patients with a different pathogen in week 2 tracheal aspirates (P-HAP group), and patients with a persistent pathogen presence in week 2 tracheal aspirates (P-P group).

### Data Analysis

Statistical analyses were carried out using R (version 4.1.1; R Foundation for Statistical Computing). Nominal variables are presented with frequencies (percentages) and numerical variables with means (SDs) or medians (IQRs) when the normality assumption is violated. For the comparison of nominal dichotomous variables, the Fisher exact test was used. Continuous variables were first assessed for normality using the D’Agostino omnibus test. A comparison of continuous variables across groups was carried out using the Kruskal-Wallis test. Dunn post hoc test with Bonferroni correction was used to adjust for multiple comparisons. The association between average temperature in the first 48 hours and the duration of mechanical ventilation, the average maximum daily requirement for noradrenaline and the average maximum FiO_2_ in the first 7 days was evaluated using Spearman correlation. Generalized linear models were used to additionally estimate the ICU and hospital survival in association with the abovementioned risk factors. A statistically significant observation was considered at *P*<.05.

## Results

### Baseline Characteristics

**Figure 1:**
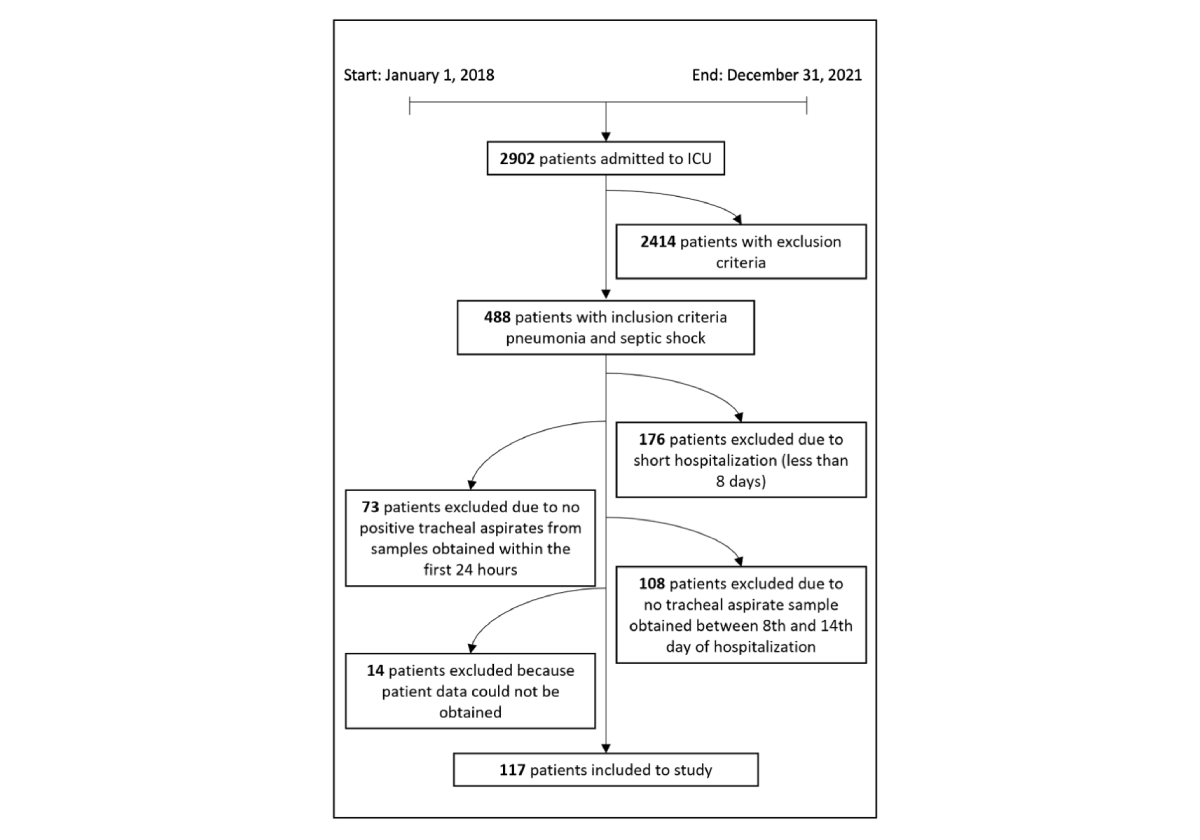
Study patient population and inclusion flowchart. ICU: intensive care unit.

In all, 117 patients were included in the study analysis; 84 (71.8%) were male, and the mean age was 63.7 (SD 13.5) years. All patients were invasively mechanically ventilated in the ICU on day 1. Mean APACHE II and SOFA scores on admission were 21.7 (SD 6.6) and 10.1 (SD 2.6), respectively. A total of 77 (65.8%) were discharged alive from the ICU, and 46 (39.3%) patients were discharged alive from the hospital. P-N, P-HAP, and P-P groups consisted of 25, 42, and 50 patients, respectively. We observed no significant differences between the P-N, P-HAP, and P-P groups in all treatment parameters apart from the use of paracetamol on day 1, which was significantly greater in the P-N group (mean 1.0, SD 1.1 g vs mean 0.4, SD 0.7 g vs mean 0.4, SD 0.8 g; *P*=.009). General demographic data and parameters describing the course of the treatment in the ICU are described in Table 1. The study patient population and inclusion flowchart are presented in Figure 1.

**Table 1:** Demographic data and parameters describing the course of treatment in the intensive care unit (ICU).

Label and variable		Group				Total		*P* value	
		P-N^a^	P-P^b^	P-HAP^c^					
Patients, n (%)		25 (21.4)	50 (42.7)	42 (35.9)	117 (100)				
**Gender, n (%)**									.26
	Female	5 (15.2)	18 (54.5)	10 (30.3)	33 (28.2)				
	Male	20 (23.8)	32 (38.1)	32 (38.1)	84 (71.8)				
**Age (years)**									.21
	Median (IQR)	62.0 (52.0-74.0)	64.0 (57.2-71.8)	67.0 (62.0-76.0)	65.0 (57.0-74.0)				
	Min-max	23.0-84.0	26.0-84.0	39.0-86.0	23.0-86.0				
**BMI (kg/m^2^)**									.19
	Mean (SD)	28.7 (7.0)	27.6 (4.3)	29.7 (6.4)	28.6 (5.8)				
	Min-max	17.2-47.7	18.8-39.2	16.3-44.8	16.3-47.7				
**Length of hospitalization (days)**									.35
	Median (IQR)	22.0 (17.0-29.0)	19.5 (15.0-26.8)	17.5 (13.0-26.8)	20.0 (14.0-26.9)				
	Min-max	8.0-53.0	10.0-43.0	8.0-69.0	8.0-69.0				
**ICU survival, n (%)**									.26
	Died	6 (15.0)	16 (40.0)	18 (45.0)	40 (34.2)				
	Survived	19 (26.1)	34 (44.2)	24 (31.2)	77 (65.8)				
**Hospital survival, n (%)**									.59
	Died	13 (18.3)	31 (43.7)	27 (38.0)	71 (60.7)				
	Survived	12 (26.1)	19 (41.3)	15 (32.6)	46 (39.3)				
**SOFA^d^ at the time of admission**									.76
	Median (IQR)	9.5 (7.0-12.0)	10.0 (9.0-12.0)	10.0 (9.0-12.0)	10.0 (9.0-12.0)				
	Min-max	5.0-16.0	5.0-15.0	4.0-15.0	4.0-16.0				
**APACHE^e^ II at the time of admission**									.53
	Mean (SD)	20.4 (6.3)	22.0 (6.4)	22.1 (7.2)	21.7 (6.6)				
	Min-max	6.0-32.0	7.0-36.0	5.0-40.0	5.0-40.0				
Change of antibiotic in 7 days after admission (no), n (%)		7 (18.4)	17 (44.7)	14 (36.8)	38 (32.5)		.86		

^a^P-N: patients with sterile week 2 tracheal aspirates.

^b^P-HAP: patients with a different pathogen in week 2 tracheal aspirates.

^c^P-P group: patients with a persistent pathogen presence in week 2 tracheal aspirates.

^d^SOFA: Sequential Organ Failure Assessment.

^e^APACHE: Acute Physiology and Chronic Health Evaluation.

### Main Results

**Figure 2:**
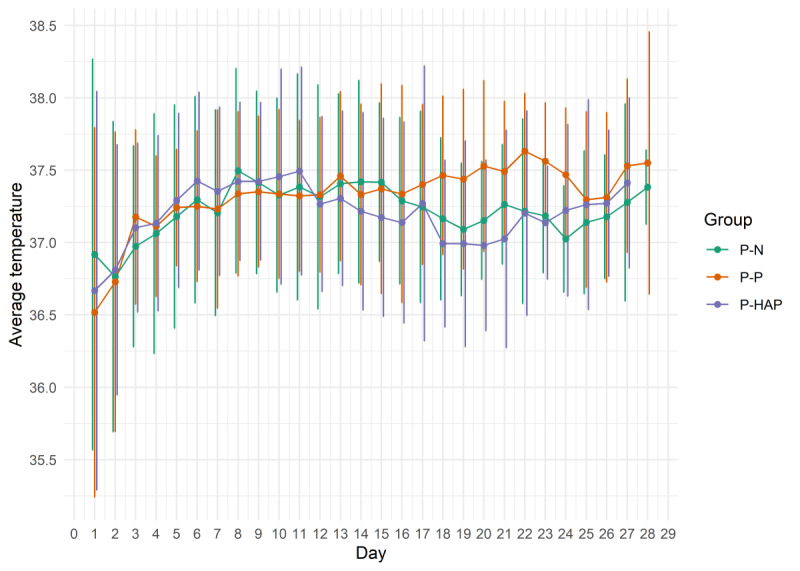
Average body temperature by groups in the first 28 days of treatment in the intensive care unit. P-HAP: patients with a different pathogen in week 2 tracheal aspirates; P-N: patients with sterile week 2 tracheal aspirates; P-P: patients with a persistent pathogen presence in week 2 tracheal aspirates.

Body temperature changes in the first 28 days of treatment are presented in Figure 2. We observed a significantly higher average temperature in the first 48 hours after admission to ICU in patients who survived to hospital discharge compared to nonsurvivors (mean 37.2 °C, SD 1 °C vs mean 36.9 °C, SD 1.6 °C; *P*=.04) and nonsignificant differences in average temperatures in the first 48 hours after admission to ICU between survivors and nonsurvivors to ICU discharge (mean 37.0 °C, SD 1.3 °C vs mean 37.0 °C, SD 1.2 °C; *P*=.60). We observed no associations between average temperatures in the first 48 hours after ICU admission and days of mechanical ventilation in the first 7 days of treatment (ρ=–0.090; *P*=.30), the average maximum daily requirement for noradrenaline in the first 7 days of treatment (ρ=–0.029; *P*=.80), average maximum FiO_2_ in the first 7 days of ICU treatment (ρ=0.040; *P*=.70), and requirement for renal replacement therapy in the first 7 days of ICU treatment (mean 37.3 °C, SD 1.4 °C vs mean 37.0 °C, SD 1.3 °C; *P*=.23).

**Figure 3:**
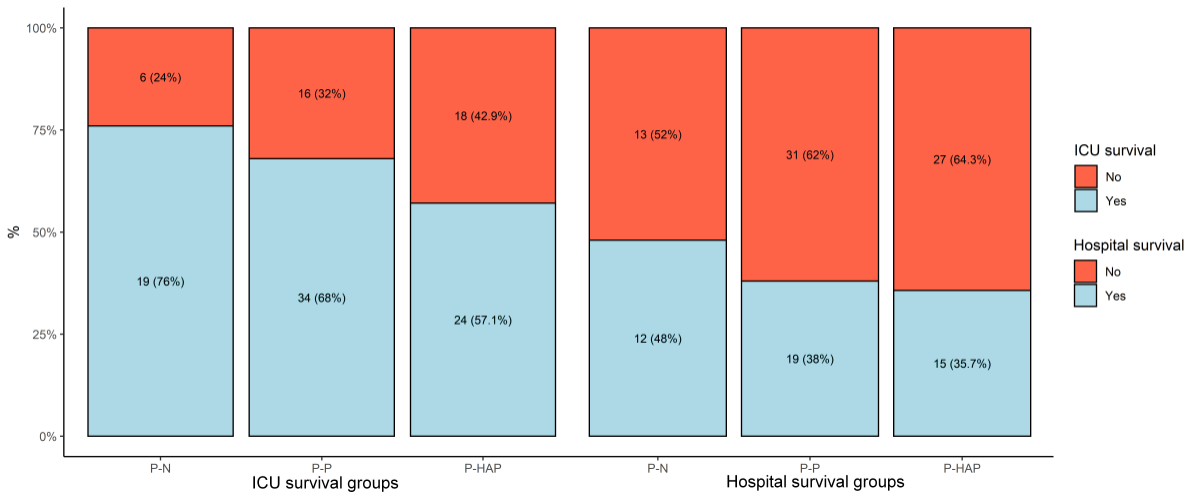
Intensive care unit (ICU) and hospital survival by groups. P-HAP: patients with a different pathogen in week 2 tracheal aspirates; P-N: patients with sterile week 2 tracheal aspirates; P-P: patients with a persistent pathogen presence in week 2 tracheal aspirates.

**Figure 4:**
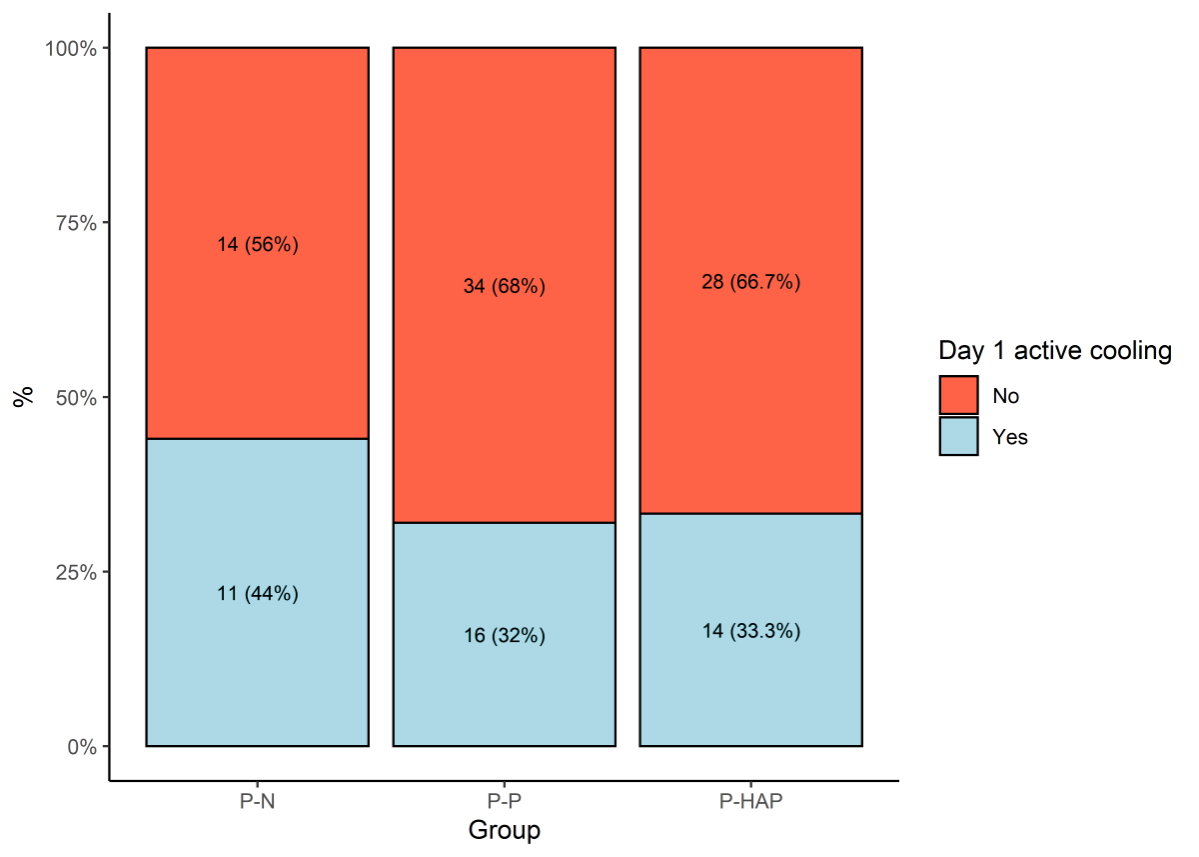
Active cooling on day one of hospitalization in the intensive care unit divided by groups. P-HAP: patients with a different pathogen in week 2 tracheal aspirates; P-N: patients with sterile week 2 tracheal aspirates; P-P: patients with a persistent pathogen presence in week 2 tracheal aspirates.

P-N, P-HAP, and P-P groups consisted of 25, 42, and 50 patients each, respectively. No significant differences in average body temperature in the first 48 hours were observed between the P-P, P-HAP, and P-N groups. Temperature variations increased after day 16 (Figure 2), however, and approximately two-thirds of patients were discharged from the ICU or died before day 16. Both hospital and ICU survival were higher in the P-N group compared to the P-HAP and P-P groups; however, the differences were not statistically significant (Figure 3). We observed statistically significant greater use of paracetamol in the first 24 hours after ICU admission in the P-N group compared to the P-HAP and P-P groups (mean 1.0, SD 1.1 g vs mean 0.4, SD 0.7 g vs mean 0.4, SD 0.8 g; *P*=.009). Active cooling was used more frequently on day 1 in the P-N group, but no significant differences were observed (Figure 4).

### Microbiological Data

In all, 148 different causal pathogens were isolated. In admission tracheal aspirates, the majority (n=124, 66%) of pathogens were gram-negative bacteria, followed by gram-positive bacteria (n=56, 29.8%) and fungal pathogens (4.3%). In week 2 tracheal aspirates, the majority (n=128, 86.5%) of pathogens were gram-negative bacteria, followed by gram-positive bacteria (n=14, 9.5%) and fungal pathogens (n=6, 4.1%). We observed no differences between the P-N, P-HAP, and P-P groups in causative pathogens in admission tracheal aspirates (Table 2). There were no differences between the P-N, P-HAP, and P-P groups in the rate of change of antimicrobial therapy within the first week after ICU admission, and we observed no changes in the duration of initial (combined empiric and antibiogram-guided antimicrobial therapy) and cumulative ICU-stay antibiotic therapy (mean 7.6, SD 2.7 days vs mean 8.4, SD 3.1 days vs mean 8.2, SD 2.9 days; *P*=.55; mean 15.2, SD 5.6 days vs mean 14.8, SD 6.8 days vs mean 15.9, SD 6.2 days; *P*=.70; respectively).

**Table 2:** Isolated pathogens from the first and second samples divided by groups.

Genus				Group, n (%)					Total, n (%)
				P-P^a^		P-HAP^b^	P-N^c^		
**Gram-positive bacteria**									
	**Sample 1**								
		*Staphylococcus*	8 (4.3)		15 (8.0)		6 (3.2)	29 (15.4)	
		*Streptococcus*	5 (2.7)		11 (5.9)		5 (2.7)	21 (11.2)	
		*Corynebacterium*	3 (1.6)		2 (1.1)		1 (0.5)	6 (3.2)	
		Total	16 (8.5)		28 (14.9)		12 (6.4)	56 (29.8)	
	**Sample 2**								
		*Staphylococcus*	5 (3.4)		2 (1.4)		N/A^d^	7 (4.7)	
		*Corynebacterium*	1 (0.7)		3 (2.0)		N/A	4 (2.7)	
		*Streptococcus*	1 (0.7)		1 (0.7)		N/A	2 (1.4)	
		*Enterococcus*	0 (0)		1 (0.7)		N/A	1 (0.7)	
		Total	7 (4.7)		7 (4.7)		N/A	14 (9.5)	
**Gram-negative bacteria**									
	**Sample 1**								
		*Klebsiella*	17 (9.0)		6 (3.2)		8 (4.3)	31 (16.5)	
		*Haemophilus*	4 (2.1)		7 (3.7)		6 (3.2)	17 (9.0)	
		*Escherichia*	9 (4.8)		4 (2.1)		1 (0.5)	14 (7.4)	
		*Enterobacter*	9 (4.8)		3 (1.6)		2 (1.1)	14 (7.4)	
		*Pseudomonas*	10 (5.3)		0 (0)		1 (0.5)	11 (5.9)	
		Other	23 (12.2)		7 (3.7)		7 (3.7)	37 (19.7)	
		Total	72 (38.3)		27 (14.4)		25 (13.3)	124 (66)	
	**Sample 2**								
		*Klebsiella*	16 (10.8)		12 (8.1)		N/A	28 (18.9)	
		*Pseudomonas*	16 (10.8)		8 (5.4)		N/A	24 (16.2)	
		*Escherichia*	11 (7.4)		4 (2.7)		N/A	15 (10.1)	
		*Enterobacter*	8 (5.4)		6 (4.1)		N/A	14 (9.5)	
		*Acinetobacter*	10 (6.8)		3 (2.0)		N/A	13 (8.8)	
		Other	18 (12.2)		16 (10.8)		N/A	34 (23.0)	
		Total	79 (53.4)		49 (33.1)		N/A	128 (86.5)	
**Fungi**									
	**Sample 1**								
		*Candida*	1 (0.5)		6 (3.2)		1 (0.5)	8 (4.3)	
		Total	89 (47.3)		61 (32.4)		38 (20.2)	188 (100)	
	**Sample 2**								
		*Candida*	5 (3.4)		1 (0.7)		N/A	6 (4.1)	
		Total	91 (61.5)		57 (38.5)		N/A	148 (100)	

^a^P-P group: patients with a persistent pathogen presence in week 2 tracheal aspirates.

^b^P-HAP: patients with a different pathogen in week 2 tracheal aspirates.

^c^P-N: patients with sterile week 2 tracheal aspirates.

^d^N/A: not applicable.

## Discussion

### Principal Findings

We observed higher initial body temperatures in patients who survived to hospital discharge and a trend toward higher initial temperatures in patients who survived to ICU discharge. We did not observe any differences in body temperature between the P-N, P-HAP, and P-P groups of patients, but we did observe a significantly greater use of paracetamol in the P-N group of patients and a trend toward greater use of active cooling in the P-N group of patients. Additionally, we observed a trend toward better survival to ICU and hospital discharge in the P-N group of patients.

Traditionally, antipyretic therapy (mostly paracetamol), and in some cases active cooling, have been used to lower body temperature in patients who are febrile with infection. The presumed patient benefit comes from decreased metabolic demands associated with lower body temperature [7,8].

The survival of our patients is comparable to studies performed by Gursel and Demirtas [9], Depuydt et al [10], and Qiao et al [11] who reported survival to ICU discharge of patients who were critically ill with pneumonia in the range between 46% and 75%, compared to 65.8% survival to ICU discharge in our patients. They included patients with similar severity of illness, with SOFA scores in the range between 4 and 6 points and APACHE II scores around 20 points, compared to the SOFA score 10.1 (SD 2.6) and APACHE II score 21.7 (SD 6.6) in our study.

Our findings are in line with a number of other studies where better survival was observed in patients with infection and physiological-grade fever (ie, body temperature between around 37 °C and 39 °C). Both Kushimoto et al [12] in a prospective observational study and Shimazui et al [13] in a retrospective observational study observed higher mortality in patients with sepsis and lower body temperature on admission. Lee et al [14] also observed lower mortality in patients with physiological-grade fever in their prospective study. Rumbus et al [15] performed a meta-analysis of 42 studies reporting body temperature and mortality in patients with sepsis. They discovered a correlation between higher body temperature and lower mortality; higher body temperature predicted better survival, and hypothermia predicted lower survival. Similar to our findings the number of patients with body temperature over 39.5 °C was very low. Thomas‑Rüddel et al [16] performed a secondary analysis of a large data set of patients with sepsis and discovered that initial body temperatures were distributed in two peaks: a smaller peak at around 35.5 °C and a larger peak approximately twice as large at around 38 °C. Again, the highest survival rate was observed in patients with hyperthermia and the lowest in patients with hypothermia. They also observed that ambient temperatures were significantly associated with body temperatures; lower ambient temperatures were associated with hypothermia, and higher outside temperatures were associated with hyperthermia. The potential benefits of active warming of patients with infection were further highlighted by Drewry et al [4], who performed a pilot randomized controlled study evaluating the use of therapeutic hyperthermia in patients with sepsis and observed a significantly better survival in the hyperthermia group; however, there were no differences in the primary results.

We can speculate that higher doses of paracetamol and greater use of active cooling probably decreased body temperatures in the P-N group of patients; however, this was not associated with any improvement during ICU treatment. We observed no differences in the use of noradrenalin, parameters of mechanical ventilation, requirement for renal replacement therapy, or organ dysfunction scores, and we observed a trend toward greater ICU and hospital survival in the P-N group. Our results suggest that there is probably no clinical benefit associated with the treatment of physiological-grade fever. Similarly, Zhang et al [17] and Ye et al [18] performed retrospective studies on patients with fever and sepsis, and they observed no beneficial effects of antipyretic therapy and possible harm associated with the use of external cooling. In a prospective randomized controlled trial, Young et al [19] observed approximately 0.5 °C lower body temperature in patients who received paracetamol (4 g daily) compared to patients who received a placebo. They also observed that patients who received more paracetamol experienced a longer ICU stay if they were nonsurvivors, and shorter ICU stay if they were survivors, which was explained by the probable effect of lower body temperature on clinicians’ perception of the patients’ prognosis. Lower body temperature in the paracetamol group was not associated with any improvement in the ICU course of treatment parameters [19,20].

To our knowledge, there is no other data to compare our results regarding the clearance or persistence of pathogens in tracheal aspirates. In 42.7% (n=50) of our patients (ie, the P-P group of patients), the same pathogen persisted in tracheal aspirates 1 week after initial samples were obtained. There were no differences in causative pathogens between the P-N, P-HAP, and P-P groups of patients, and we observed no significant changes in baseline data between the groups, including the rate of change of empiric antibiotic therapy. There was a trend toward higher temperature in the first 48 hours in the P-N group, compared to the P-HAP and P-P groups, possibly indicating that tolerance of hyperthermia could be beneficial for patients who are intubated with pneumonia and sepsis. There were no significant differences in the course of ICU treatment parameters despite greater use of paracetamol and active cooling, indicating that pharmacological or active cooling has no benefit in patients where fever is part of an appropriate response to infection.

There are a number of limitations to our study. We performed a single-center retrospective observational study with all inherent biases associated with this study design. Of the initial 488 patients with pneumonia, 249 were not included because there were no tracheal aspirates 1 week after ICU admission (because of extubation, death, or no clinical need for obtaining samples if the patients were still intubated). Additionally, target temperatures were defined by the treating physicians. However, there are no guidelines regarding target temperatures for this patient population, and we observed no differences in baseline data between the different groups.

### Conclusions

To conclude, we observed better survival in patients who developed higher body temperatures in the first 48 hours after admission to the ICU; however, we observed no changes in other treatment parameters. Additionally, we observed greater use of interventions aimed at cooling the patients (use of paracetamol and a trend toward greater use of active cooling) in patients with negative tracheal aspirates 1 week after ICU admission. Additionally, in this group of patients, we observed a trend toward better survival. Our results speak against the use of interventions aimed at the reduction of body temperature and support the strategy of temperature tolerance in patients who are mechanically ventilated with pneumonia and sepsis.

## Abbreviations

APACHE: acute physiology and chronic health evaluation
ICU: intensive care unit
P-HAP: patients with a different pathogen in week 2 tracheal aspirates
P-N: patients with sterile week 2 tracheal aspirates
P-P: patients with a persistent pathogen presence in week 2 tracheal aspirates
SOFA: sequential organ failure assessment
